# Symplasmic isolation marks cell fate changes during somatic embryogenesis

**DOI:** 10.1093/jxb/eraa041

**Published:** 2020-01-24

**Authors:** Kamila Godel-Jedrychowska, Katarzyna Kulinska-Lukaszek, Anneke Horstman, Mercedes Soriano, Mengfan Li, Karol Malota, Kim Boutilier, Ewa U Kurczynska

**Affiliations:** 1 Institute of Biology, Biotechnology and Environmental Protection, Faculty of Natural Sciences, University of Silesia in Katowice, Katowice, Poland; 2 Bioscience, Wageningen University and Research, AA Wageningen, Netherlands; 3 Laboratory of Molecular Biology, Wageningen University and Research, AA Wageningen, Netherlands; 4 Institute of Biology, Biotechnology and Environmental Protection, Faculty of Natural Sciences, University of Silesia in KatowiceKatowice, Poland; 5 Royal Holloway, University of London, UK

**Keywords:** Arabidopsis, auxin, *BABY BOOM*, plasmodesmata size exclusion limit, plasmodesmata, somatic embryogenesis, symplasmic communication, symplasmic domain, *WOX2*

## Abstract

Cell-to-cell signalling is a major mechanism controlling plant morphogenesis. Transport of signalling molecules through plasmodesmata is one way in which plants promote or restrict intercellular signalling over short distances. Plasmodesmata are membrane-lined pores between cells that regulate the intercellular flow of signalling molecules through changes in their size, creating symplasmic fields of connected cells. Here we examine the role of plasmodesmata and symplasmic communication in the establishment of plant cell totipotency, using somatic embryo induction from Arabidopsis explants as a model system. Cell-to-cell communication was evaluated using fluorescent tracers, supplemented with histological and ultrastructural analysis, and correlated with expression of a *WOX2* embryo reporter. We showed that embryogenic cells are isolated symplasmically from non-embryogenic cells regardless of the explant type (immature zygotic embryos or seedlings) and inducer system (2,4-dichlorophenoxyacetic acid or the BABY BOOM (BBM) transcription factor), but that the symplasmic domains in different explants differ with respect to the maximum size of molecule capable of moving through the plasmodesmata. Callose deposition in plasmodesmata preceded *WOX2* expression in future sites of somatic embryo development, but later was greatly reduced in *WOX2*-expressing domains. Callose deposition was also associated with a decrease *DR5* auxin response in embryogenic tissue. Treatment of explants with the callose biosynthesis inhibitor 2-deoxy-D-glucose supressed somatic embryo formation in all three systems studied, and also blocked the observed decrease in *DR5* expression. Together these data suggest that callose deposition at plasmodesmata is required for symplasmic isolation and establishment of cell totipotency in Arabidopsis.

## Teaser

The switch from somatic to totipotent growth is marked by the establishment of distinct embryogenic symplasmic domains and requires callose deposition at plasmodesmata.

## Introduction

Intercellular communication between plant cells takes place by the apoplastic pathway, in the shared space within cell walls, or by the symplasmic pathway through plasmodesmata (PD) that traverse the walls of adjacent cells and connect their cytoplasm. PD are plasma membrane-lined channels containing a central tube of endoplasmic reticulum (desmotubule) that connects adjacent cells (Tilsner *et al.*, 2016). PD provide intercellular transport routes not only for small molecules such as water and nutrients, but also for signalling molecules, such as hormones, small RNAs, and transcription factors, and for viruses (Ding *et al.*, 1992; Haywood *et al.*, 2002; Kurata *et al.*, 2005; Ueki and Citovsky, 2005; Han *et al.*, 2014; Kitagawa and Jackson, 2017; Yuan *et al.*, 2017; Kehr and Kragler, 2018). PD control the cell-to-cell flow of molecules, and can be reduced or closed through deposition of callose (β-1,3-glucan) in the neck region (Benitez-Alfonso *et al.*, 2013; Han *et al.*, 2014). The level of callose deposition is regulated by a balance between callose synthase and β-1,3-glucanase activity (Zavaliev *et al.*, 2011; de Storme and Geelen, 2014; Sevilem *et al.*, 2015). Callose deposited in the cell wall serves to restrict the flow of molecules through PD by decreasing the size exclusion limit (SEL). Callose degradation by β-1,3-glucanases increases cell-to-cell movement of molecules by increasing the SEL (Wu *et al.*, 2018). A change in PD SEL by callose deposition alters PD permeability and occurs in response to both internal and external factors (Chen and Kim, 2009; Simpson *et al.*, 2009; Cui and Lee, 2016; Amsbury *et al.*, 2017). Callose turnover at PD is an important mechanism regulating movement of signalling molecules during development (Sevilem *et al.*, 2015; Saatian *et al.*, 2018), including shoot apical meristem development (Rinne *et al.*, 2011), lateral root formation (Benitez-Alfonso *et al.*, 2013), stomata patterning (Guseman *et al.*, 2010), root nodulation (Gaudioso-Pedraza *et al.*, 2018), and pollen development (Li *et al.*, 2003)

PD traverse cell walls to establish a symplasmic continuum, but groups of cells that are interconnected by functional PD can also be separated from surrounding cells through the absence or modification of PD, thus forming permanent or temporary symplasmic domains (Erwee and Goodwin, 1985; Lucas *et al.*, 1993; Rinne and van der Schoot, 1998; Ehlers and Kollamnn, 2001; Otero *et al.*, 2016; Wu *et al.*, 2016). It has been proposed that (temporary) symplasmic isolation is a universal prerequisite for cell (re)differentiation (Ehlers and van Bel, 1999). Symplasmically connected cells usually divide with the same frequency and in the same direction (Ehlers and Kollmann, 2000), whereas changes in the PD SEL, PD number or PD functionality that result in decreased cell-to-cell connections between groups of cells are associated with changes in cell fate, the formation of new structures, and cell differentiation (for reviews see Burch-Smith and Zambryski, 2016; Tilsner *et al.*, 2016). During embryogenesis, movement of molecules through PD is progressively restricted with more advanced stages of embryo development, and is correlated with organ and tissue differentiation (Kim *et al.*, 2002; Kim and Zambryski, 2005). In Arabidopsis roots, changes in symplasmic communication are associated with both the initiation and positioning of lateral root meristems (Benitez-Alfonso *et al.*, 2013), while loss of symplasmic signalling to and from the Arabidopsis root endodermis results in an increased number of endodermis cell layers and misspecification of the stele (Wu *et al.*, 2016). A decrease in symplasmic movement through PD is essential for correct stomatal patterning during epidermis development (Guseman *et al.*, 2010). These examples illustrate that symplasmic cell-to-cell communication is one of the mechanisms that plants use to control their growth and development.

Plant tissues are developmentally flexible and can be induced to regenerate *in vitro* in response to plant growth regulator or stress treatments. *In vitro* regeneration takes place through embryo formation from totipotent cells or through successive organ formation from pluripotent cells (Rocha *et al.*, 2015; Yu *et al.*, 2017). Somatic embryogenesis (SE) is an expression of plant cell totipotency, in which embryos develop from vegetative tissues, rather than from the zygote. SE can be induced by treating explants with the synthetic auxin 2,4-diclorophenoxyacetic acid (2,4-D) (Fehér *et al.*, 2003; Raghavan, 2004), but also by ectopic overexpression of a number of plant transcription factors, including AINTEGUMENTA-LIKE (AIL) AP2/ERF proteins such as BABY BOOM (BBM) (Horstman *et al*., 2017*a*,*b*). Somatic embryo development and organogenesis often occur side by side in the same explant (Boutilier *et al.*, 2002; Raghavan, 2004; Bassuner *et al.*, 2007), but can be distinguished at an early stage at the cellular and gene expression levels. In thin sections, totipotent (embryogenic) cells can be distinguished from pluripotent (meristematic) cells by their relatively larger euchromatic nucleus with a single large nucleolus, compared with pluripotent cells, which have a relatively small, heterochromatic nucleus with one or more nucleoli (Verdeil *et al.*, 2007). A number of well-characterized embryo reporter lines are available that accurately distinguish totipotent cells from pluripotent cells (Gaj *et al.*, 2005; Li *et al.*, 2014; Zhou *et al.*, 2017; Kadokura *et al.*, 2018).

There are only a few studies on PD in explants undergoing SE (Dubois *et al.*, 1991; Canhoto *et al.*, 1996; Puigderrajols *et al.*, 2001; Verdeil *et al.*, 2001; Grimault *et al.*, 2007; Reis *et al.*, 2008). Callose was observed in the cell walls of embryogenic cells and young embryos in embryogenic cultures of chicory, coconut, and cork oak, but not during later embryo growth, suggesting that initial physical and physiological isolation of embryogenic cells is necessary to initiate SE (Dubois *et al*., 1990, 1991; Puigderrajols *et al.*, 2001; Verdeil *et al.*, 2001; Grimault *et al.*, 2007). This role for symplasmic isolation during somatic embryo initiation was inferred primarily from ultrastructural analysis of PD or from the presence of callose, but such studies do not provide direct support for symplasmic isolation, as information on the functionality of PD is lacking. By contrast, the movement of symplasmic tracers such as the low molecular mass fluorochromes, fluorescein isothiocyanate-conjugated dextran (F-dextran) or green fluorescent protein (GFP) can be tracked within a tissue or organ to identify symplasmically connected or isolated areas (Duckett *et al.*, 1994; Kim *et al.*, 2005; Stadler *et al.*, 2005; Kragler, 2015).

Here we used fluorescent tracers in combination with fluorescent embryo reporter lines to study the role of symplasmic isolation during 2,4-D- and BBM-induced SE. Our results show that the explant regions engaged in SE are symplasmically isolated, regardless of the experimental system, and that callose biosynthesis is required for somatic embryo initiation and outgrowth. Together, these data support the idea that symplasmic isolation and directional flow of molecules are required for and mark cell fate reprogramming to SE.

## Materials and methods

### Plant material and culture conditions

The following Arabidopsis (L.) Heynh Columbia-0 (Col-0) lines were used for *in vitro* culture: wild-type (WT), *35S:BBM* (Boutilier *et al.*, 2002), *35S:BBM WOX2:NLS-YFP* (Breuninger *et al.*, 2008), and *35S:BBM-GR Dr5v2tdTomato* (Horstman *et al.*, 2017*b*; Liao *et al.*, 2015). All culture procedures have been described previously. Somatic embryo cultures were initiated from immature zygotic embryos (IZEs) cultured on modified B5 solid medium (Gamborg *et al.*, 1968) supplemented with 5 μM 2,4-D (Sigma-Aldrich; Gaj, 2001). For *35S:BBM* plants, somatic embryo cultures were initiated from IZEs, as described above, but in medium lacking 2,4-D, or from germinating seeds on basal medium (Horstman *et al.*, 2017*b*). Activation of the BBM–glucocorticoid receptor (GR) fusion protein was performed using 10 µM dexamethasone as in Horstman *et al.* (2017*b*).

### Histological analyses

Processing of explants for stereo- and bright field microscopy was performed as in Sala *et al.* (2017). Sections were stained with 0.1% toluidine blue O (Sigma-Aldrich) in phosphate-buffered saline and examined under an Olympus BX45 microscope equipped with an Olympus XC50 digital camera.

### Analysis of symplasmic tracer distribution

Fluorescein bis-(5-carboxymethoxy-2-nitrobenzyl) ether, dipotassium salt (CMNB-caged fluorescein; Thermo Fisher Scientific) was prepared and detected as described earlier (Wrobel *et al.*, 2011). Fluorescein was uncaged in different parts of the explants at different stages of development. The spatial pattern of fluorescein distribution was monitored immediately after uncaging and at the time points indicated in the text.

8-Hydroxypyrene-1,3,6-trisulphonic acid trisodium salt (HPTS; Sigma-Aldrich) and F-dextran (molecular mass 3 kDa; Sigma-Aldrich) were prepared in liquid half-strength Murashige and Skoog (½MS) medium at 5 mg ml^−1^. To monitor movement of HPTS and F-dextran, the explants were injured with a microcapillary and immersed in the fluorescent tracer solution, or injured with a microcapillary previously filled with the tracer solution. The explants were pretreated with a 0.1 mM solution of 2-deoxy-D-glucose (DDG; Sigma-Aldrich, D8375) in ½MS medium for 30 min to prevent wound-induced callose production. The conditions for excitation and detection of HPTS and uncaged fluorescein were described previously (Wróbel-Marek *et al.*, 2017).

### 2-Deoxy-D-glucose treatment

DDG (Radford *et al.*, 1998) was dissolved in demineralized water. A 0.1 μM solution was applied in the form of two to three droplets on the explant surface, which was then cultured on the same medium as described above. Explants were treated with DDG for 7 d (DDG was refreshed daily), and then transferred to medium without DDG for further development. A 7 d DDG treatment was chosen as it corresponds to the period in which SE is initiated. SE was evaluated after 7 and 12 d of culture. The number of embryogenic protrusions and somatic embryos was visually determined using a stereo microscope.

### Ultrastructural analysis and three-dimensional reconstruction of plasmodesmata

Samples were prepared for array tomography (AT) analysis as described by Milewska-Hendel *et al.* (2017). Sections 130 nm thick were cut with an advanced substrate holder (ASH-100, RMC Boeckeler) using a Leica EM UC6 ultramicrotome, placed on a silicon wafer, stained with a saturated solution of uranyl acetate (Polysciences, Germany) in 50% ethanol for 15 min and 0.4% lead citrate agents (Sigma-Aldrich, Poland) for 10 min. Image stacks were collected using an Apreo scanning electron microscope with 4 nm per pixel resolution. Manual segmentation of cells was carried out in Microscope Image Browser (MIB) software (GNU General Public License v2; Belevich *et al.*, 2016). Three-dimensional (3-D) models of cells and structures were generated after segmentation, and images were made using Amira Software (trial version, Thermo Fisher Scientific).

The average number of PD between totipotent cells, pluripotent cells and between totipotent and pluripotent cells in IZE explants was counted on the fifth day of culture. PD frequency (*F*) was calculated according to Ma and Peterson (2001) with the formula *F*=*N*/[*L*(*T*+1.5*R*)], where *N* is the number of PD along the wall, *L* is the length of analysed wall, *T* is the thickness of sections (0.13 µm), and *R* is the PD radius. PD were counted in three independent samples, in five cells per sample in each symplasmic domain.

### Reporter analysis

*WOX2:NLS-YFP* expression was detected using confocal laser scanning microscopy (CLSM; Olympus FV1000; excitation at 488 nm and emission detected at 500–600 nm). *DR5v2:tdTomato* expression was examined using epifluorescence microscopy (Nikon Eclipse Ni) in green light or by CLSM (excitation at 543 nm and emission detected at 555–655 nm).

### Callose staining

Callose was detected by staining for 1 h with 0.1% (w/v) aniline blue (AppliChem) in phosphate buffer (pH 7.2; Müller *et al.*, 2015). Aniline blue was observed using CLSM (excited at 405 nm and emission detected at 425–475 nm) or epifluorescence microscopy (Nikon Eclipse Ni) in UV light.

### Image processing

Images from sections were reconstructed using Corel Draw X6. CLSM images were prepared using ImageJ software. At least five optical sections were merged to one z-stack projection. The epifluorescence microscopy images were prepared using Corel Photo-Paint software (brightness and contrast were adjusted).

## Results

We examined symplasmic communication during somatic embryo induction by following the distribution of symplasmic tracer fluorochromes in three SE systems: (i) 2,4-D-induced SE from WT IZEs; (ii) *35S:BBM*-induced SE from IZE explants; and (iii) *35S:BBM*-induced SE from seedling explants. The three systems differ with respect to the explant and inducer treatments, but are similar in that somatic embryos develop directly from the explant without an intermediate callus or without further changes in the medium or culture conditions.

### Symplasmic domains are established during 2,4-D-induced somatic embryogenesis that coincide with the establishment of embryogenic cells

**Fig. 1.**
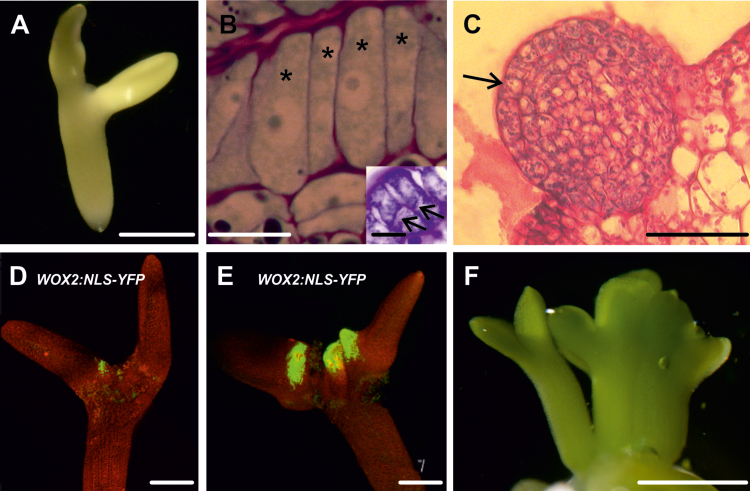
Development of WT IZE explants during 2,4-D-induced somatic embryogenesis. (A) Explant on the fifth day of culture. (B) Elongated protodermal cells (asterisks) before the first periclinal divisions. Inset, elongated cells undergoing periclinal (arrows) division. (C) Globular somatic embryo (the arrow indicates the protodermis). (D, E) *WOX2* expression in growth protrusions on the sixth day (D) and between the sixth and seventh day (E) of culture. (F) Bipolar somatic embryos formed on the IZE explant after about 3 weeks of culture. Scale bars: (A, E, F) 500 µm; (B) 100 µm; (B inset) 20 µm; (C) 200 µm; (D) 250 µm.

2,4-D-induced SE from WT Col-0 IZEs has been described previously (Gaj, 2001; Kurczyńska *et al.*, 2007) and is summarized in Fig. 1 and Supplementary Table S1. In this system, somatic embryos develop directly on the proximal adaxial region of the IZE cotyledons (Fig. 1A) from periclinal divisions of elongated protodermal cells (Fig. 1B). Cells predestined to elongate exhibit a dense cytoplasm and a large nucleus with a single large nucleolus. Globular-shaped somatic embryos with a protoderm develop after about 14 d of culture (Fig. 1C). Analysis of *WOX2:NLS-YFP* IZEs during different points of the culture showed that *WOX2* gene expression correlates with explant areas engaged in SE and the formation of somatic embryos (Fig. 1D, E). Bipolar embryos with cotyledons and a root pole were observed on the explants after 3 weeks of culture (Fig. 1F).

**Fig. 2.**
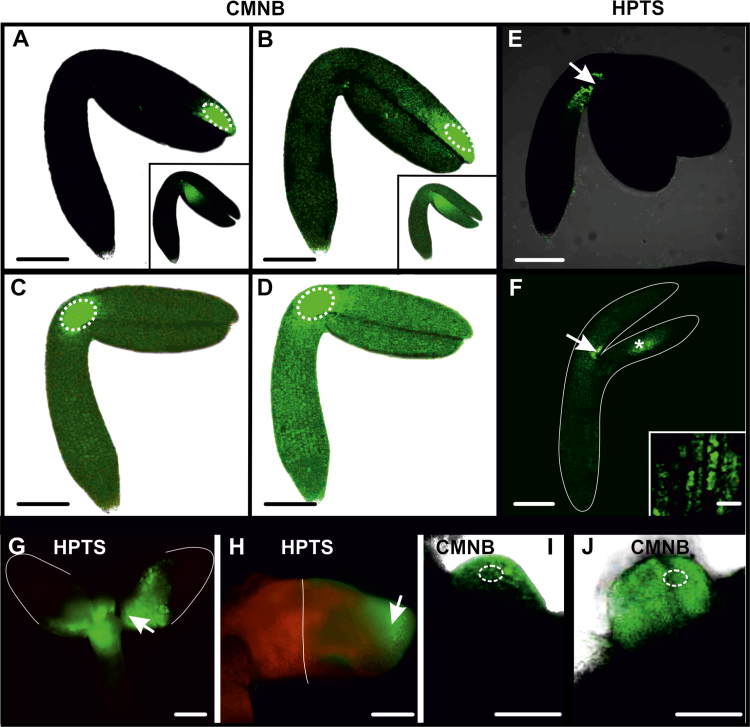
Symplasmic communication in WT IZE explants cultured on 2,4-D. (A) Freshly isolated IZE at the start of culture, 10 min after uncaging of CMNB-caged fluorescein in the distal part of cotyledon. Fluorescence is visible in a few cells next to the activation site (dotted white ellipses mark the area where CMNB was uncaged, arrows point to site of HPTS application). The inset shows a similar fluorochrome distribution when uncaging was performed in a different explant area. (B) The same IZE, 30 min after fluorescein uncaging. Weak fluorescence is observed in the entire explant. The inset shows a similar fluorochrome distribution when uncaging was performed in a different explant area. (C) Explant after 1 d of culture, 5 min after fluorescein uncaging in the basal part of cotyledon. Weak fluorescence is visible in the entire explant. (D) Explant from (C), 20 min after fluorescein activation. Distinct fluorescence is visible in the whole explant. (E) Freshly isolated IZE, 20 min after HPTS treatment. Fluorescence is visible at the site of application. (F) One-day-old explant, 20 min after HPTS treatment. Intense fluorescence at the place of application and weaker fluorescence throughout the rest of the explant. The white line indicates the outline of the explant, as seen in bright field. The inset is a magnified view of the area marked by an asterisk showing the presence of fluorochrome inside the cells. (G) HPTS applied to the proximal part of cotyledons at day 7 is not transported to the distal cotyledon (2 h after application). The white line outlines the border of the explant. (H) HPTS applied on the distal part of cotyledon at day 7 is not transported to the proximal region (2 h after application). The white line demarcates the embryogenic and non-embryogenic areas of the explant. (I) Fluorescence 20 min after uncaging CMNB within the embryogenic protrusions (12 d of culture). (J) Fluorescence 30 min after uncaging CMNB in the globular somatic embryo (12 d of culture). Images (A–F) were collected by CLSM and images (G–J) were collected by fluorescence microscopy. Scale bars: (A–F, A inset, B inset) 100 µm; (G–J) 50 µm; (F inset) 10 µm.

We examined the behaviour of two fluorescent tracers in 2,4-D-treated IZEs, CMNB-caged fluorescein and HPTS. The use of two different fluorochromes was dictated by (i) their different molecular masses (uncaged CMNB, 332 Da; HPTS, 520 Da) and diameters (uncaged CMNB, 0.4 nm; HPTS, 0.9 nm); and (ii) the possibility to differentiate between sites of application/uncaging, which increased the ability to analyse precisely the movement of fluorochromes between different explant areas. Both tracers were observed from the start of culture (freshly isolated explants) until the appearance of somatic embryos. In freshly isolated explants, both tracers remained close to the site of uncaging/application, followed later by weak fluorescence that was observed throughout the explant irrespective of the uncaging/application (Fig. 2A, B, E). Similar results were obtained in 1-day-old explants when CMNB or HPTS was used; however, tracer movement was faster in comparison to freshly isolated IZE explants (Fig. 2C, D, F). Thus, the initial slow movement of fluorochromes within explant cells in freshly isolated IZE explants is enhanced during culture with 2,4-D. Moreover, the observation that fluorochrome movement did not depend on the site of uncaging/application indicates that at this stage of culture the explant comprises a single symplasmic domain.

Symplasmic transport was maintained throughout the entire explant at the same level up to the sixth day of culture, at which point it became more restricted. This restriction in fluorochrome movement coincided with cotyledon swelling, the initiation of somatic embryo formation, and *WOX2* expression (Fig. 1; Kurczyńska *et al.*, 2007). When HPTS or CMNB caged fluorescein was applied to the cotyledon node, fluorescence was visible in the proximal, but not in the distal, part of the explant cotyledons and hypocotyl (Fig. 2G). Likewise, when the same fluorochromes were uncaged/applied to the distal part of the cotyledons, the fluorescence signals remained where they were applied (Fig. 2H). Together these data suggest that at this stage of development, embryogenic (cotyledon node) and non-embryogenic (shoot apical meristem, distal part of cotyledons and hypocotyl) explant domains were symplasmically isolated. CMNB uncaging in the embryogenic centres or in emerging somatic embryos of older explants resulted in the retention of the fluorochrome in these cells (Fig. 2I, J). The above results indicate that changes in symplasmic communication occurred during somatic embryo culture, with the result that embryogenic domains and developing embryos within the explant became symplasmically isolated from the non-embryogenic domains (Table 1).

**Table 1.** Quantification of dye movement between embryogenic and non-embryogenic regions after uncaging/application of fluorochromes and 3 kDa dextran in different Arabidopsis explants at 7 d after culture

Cell identity/ symplasmic domain	% movement from embryogenic to non-embryogenic areas			% movement from non-embryogenic to embryogenic areas		
	CMNB (*M*=332 Da)	HPTS (*M*=520 Da)	Dextran (*M*=3 kDa)	CMNB (M=332 Da)	HPTS (*M*=520 Da)	Dextran (*M*=3 kDa)
WT IZEs on 2,4-D	4.9±11.3^a^ (*n*=40)	2.7±7.6^a^ (*n*=38)	0±0^c^ (*n*=25)	5.5±7.6^a^ (*n*=38)	0±0^c^ (*n*=40)	0±0^c^ (*n*=25)
*35S:BBM* IZEs	4.8±6.4^a^ (*n*=42)	6.6±8.8^a^ (*n*=27)	0±0^c^ (*n*=19)	2.2±9.1^a^ (*n*=41)	0±0^c^ (*n*=32)	0±0^c^ (*n*=19)
*35S:BBM* seedlings	100±0^b^ (*n*=37)	100±0^b^ (*n*=27)	2.8±7.6^a^ (*n*=34)	100±0^b^ (*n*=43)	100±0^b^ (*n*=27)	0±0^c^ (*n*=35)

The data are the mean ±SE of three biological replicates; *n* is the total number of areas where the tracer was uncaged/applied. % movement=(number of areas where the tracer moved/total number of areas where the tracer was uncaged/applied)×100; 0% movement indicates that cells following different developmental fates are symplasmically isolated; low % movement indicates very little movement/symplasmic communication between cells following different developmental fates; 100% movement indicates that cells following different developmental fates are highly symplasmically connected. A *z*-test for significance between percentage values was used to determine whether there are statistically significant differences between movement of tracers with different molecular masses between areas realizing different developmental programmes, within and between experimental systems (WT IZEs, *35S:BBM* IZEs, and *35S:BBM* seedlings). Each value was compared pairwise simultaneously and statistically significant differences (*P*<0.05) between values are indicated by different letters.

### Different symplasmic domains mark embryogenic and non-embryogenic cell fates in BBM IZE explants

**Fig. 3.**
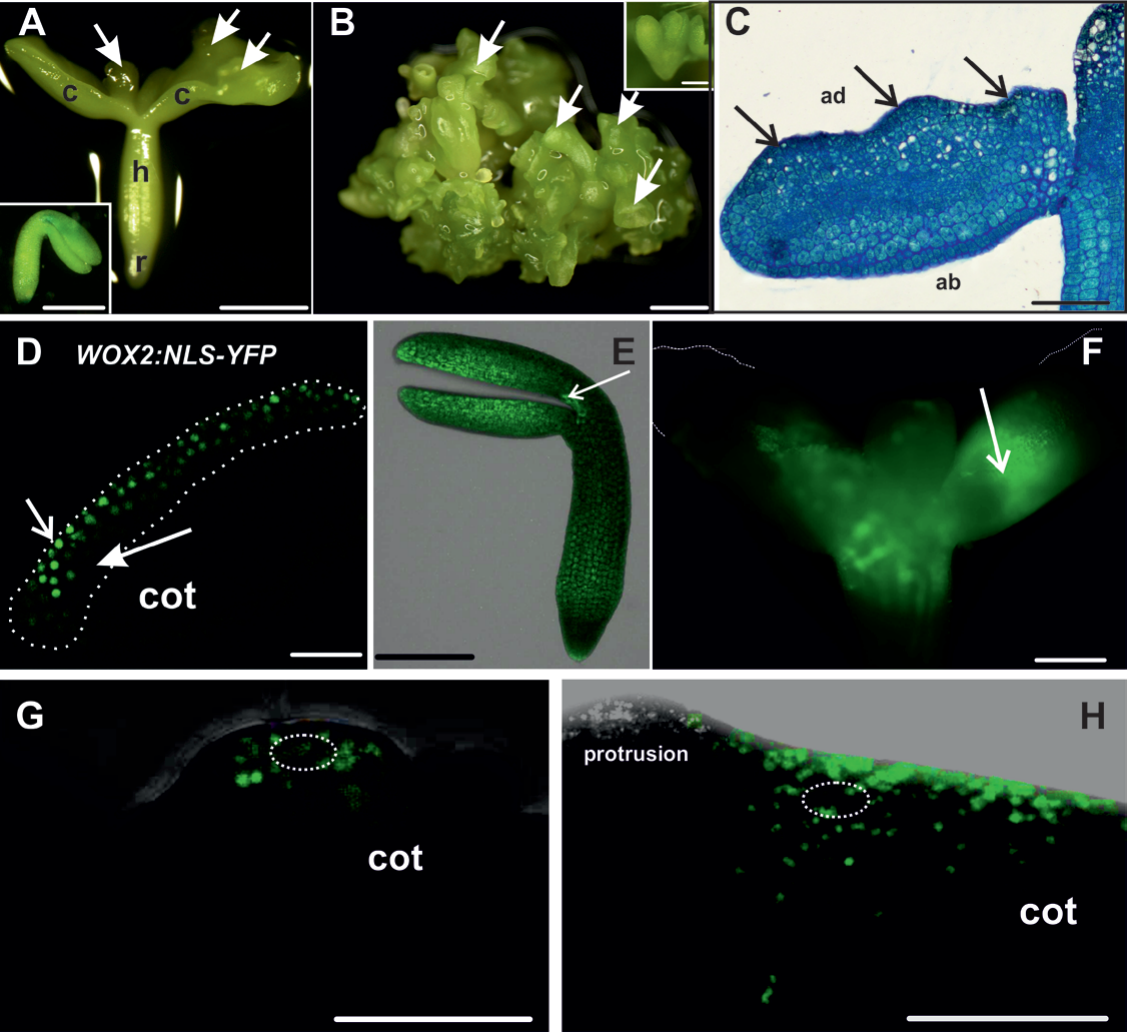
Development and symplasmic communication of cultured *35S:BBM* IZE explants. (A) Explant at day 10. Embryos appear along the length of the cotyledons (arrows; c, cotyledon; h, hypocotyl; r, root). Inset, explant morphology at the beginning of the culture. (B) Groups of somatic embryos (arrows) after 28 d of culture; secondary somatic embryos form on the primary embryos (inset). (C) Explant after 4 d of culture; arrows indicate protrusions on the adaxial (ad) side of the cotyledons (ab, abaxial). (D) Expression of *WOX2:NLS-YFP* in a few layers of protodermal and subprotodermal cells in the adaxial side of a 3-day-old explant cotyledon (cot). The dotted white line demarcates the area engaged in SE. (E) One-day-old explant, 30 min after applying HPTS; the explant is still a single symplasmic domain. The white arrow indicates the site of fluorochrome application. (F) Seven-day-old explant, 30 min after HPTS application on the cotyledon node. The white arrow indicates the site of fluorochrome application. The fluorochrome moved through the explant, with the exception of the distal parts of cotyledons. (G) Six-day-old explant after CMNB uncaging in an embryogenic protrusion on the adaxial side of cotyledon (cot). The dotted white ellipse marks the uncaging area. (H) Six-day-old explant after CMNB uncaging in a non-protruding (non-embryogenic) region of the explant. The dotted white ellipse marks the uncaging area. Scale bars: (A, A inset, B, B inset) 500 µm; (C, E, F) 100 µm; (D, G, H) 50 µm.

SE from *35S:BBM* immature IZE explants (Fig. 3; Supplementary Table S1) has not been described previously. In this system, somatic embryos at different developmental stages were clearly visible after 2 weeks of culture (Fig. 3A, B), followed shortly thereafter by secondary SE from the primary somatic embryos (Fig. 3B). During the first few days of culture, the explants increased in size and growth protrusions were observed along the adaxial side of the cotyledons (Fig. 3C). Unlike 2,4-D-treated WT IZE explants, in which thickening occurs on the proximal part of the cotyledon and embryos develop from protodermal cells, *35S:BBM* IZE cotyledons thickened over their entire length (Fig. 3C) and embryogenic cells originated from the epidermal and subepidermal cell layers (Fig. 3C). *WOX2* expression coincided with visibly embryogenic areas (Fig. 3D).

Cell-to-cell communication was also examined in *35S:BBM* IZE explants. In freshly isolated explants, HPTS fluorescence was observed throughout the whole explant within 20 min after application, and in 1-day-old explants the intensity of HPTS fluorescence increased and spread throughout the whole explant after 15 min of application (Fig. 3E), indicating that *35S:BBM* IZEs comprise a single symplasmic domain at the beginning of the culture. After 4 d of culture, two distinct symplasmic domains were detected in the explants after HPTS application at the cotyledon node: a domain with high tracer fluorescence at the cotyledon node and proximal regions of the cotyledons and hypocotyl (Fig. 3F), and a domain at the distal part of cotyledons where the tracer was excluded (Fig. 3F). The more advanced the SE culture, the more limited the areas of individual symplasmic domains became (Fig. 3G, H). CMNB-caged fluorescein/HPTS tracer remained in the area of the explant where it was uncaged/applied, that is, it did not move from embryogenic to non-embryogenic regions of the explant or vice versa (Fig. 3G, H). The results indicate that, as with WT IZE explants, *35S:BBM* IZE explants initially comprise a single symplasmic domain for low-molecular-mass compounds, but later, symplasmically isolated domains are formed where embryogenic cells develop on the explant (Table 1). This suggests that the PD SEL decreases on the border of the embryogenic and non-embryogenic domains.

### Embryogenic regions of *35S:BBM* seedlings are symplasmically isolated, but with higher size exclusion limit than IZE explants

**Fig. 4.**
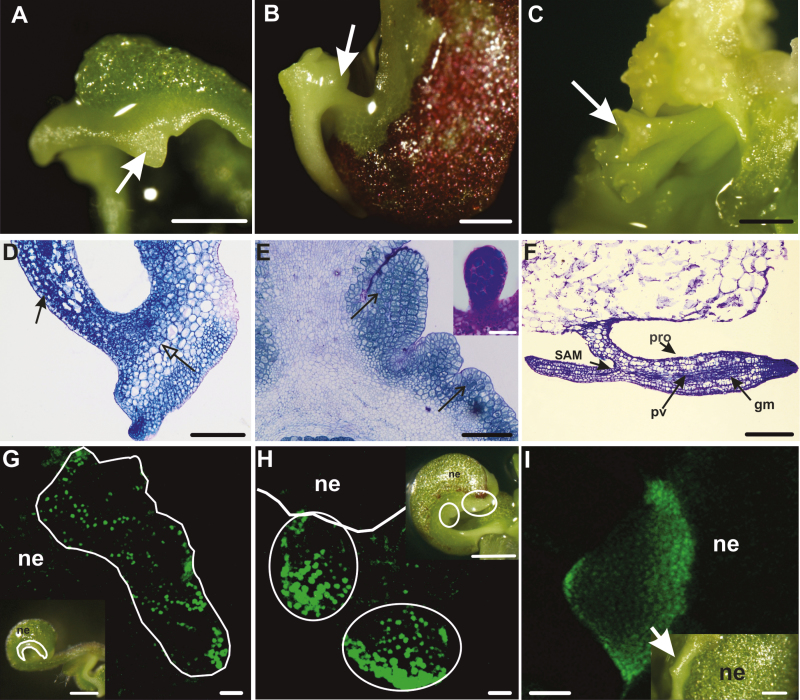
Development of cultured *35S:BBM* seedlings. (A) Dense growth on the margin of the cotyledon after 6 d of culture, from which an embryogenic protrusion (arrow) emerged. (B) Single somatic embryo at the cotyledon stage (arrow) growing at the edge of the explant cotyledon after 11 d of culture. (C) A group of somatic embryos (arrow) covering a 14 day-old explant. (D) Section of an explant cotyledon with embryogenic (closed arrow) and non-embryogenic (open arrow) regions after 5 d of the culture. (E) Section through the margin of the explant cotyledon after 9 d of culture. The arrows point to groups of cytoplasmically rich cells developing into somatic embryos (inset, somatic embryo at the globular stage of development). (F) Longitudinal section through a somatic embryo connected to the seedling by one of its cotyledons (14 d of culture; gm, ground meristem; pro, protodermis; pv, provascular tissue; SAM, shoot apical meristem). (G) Expression of *WOX2:NLS-YFP* after 5 d of culture in the cotyledon of the seedling explant (longitudinal optical section from adaxial to abaxial surface of cotyledon); different intensities of YFP expression are visible in different regions of the explant: relatively high YFP expression along the cotyledon margin where embryogenic cells form (outlined area) and decreasing YFP expression toward the deeper non-embryogenic (ne) regions of the explant. (H) Early stages of SE (7 d of culture) where embryogenic protrusions (marked by ellipses) develop on the explant. The solid white line marks the border between the embryogenic and non-embryogenic (ne) regions. (I) Expression of *WOX2:NLS-YFP* in an embryogenic protrusion within the margin. Insets in (G–I) are light images of representative seedlings. (A–C) and the insets in (G–I) are stereomicroscope images, (D–F) bright field microscope images, and (G–I) CLSM images. Scale bars: (A, B, C, G inset, H inset) 500 µm; (D, G–I) 50 µm; (E) 100 µm; (F, I inset) 200 µm.

Somatic embryos develop directly from the cotyledon margins of *35S:BBM* seedlings in the absence of inducer treatments (Boutilier *et al.*, 2002). The major steps in BBM-induced somatic embryo development from seedlings are summarized in Fig. 4. Embryogenic tissue is visible under the stereomicroscope as smooth, pale green bands that encircle the cotyledons as early as 5–7 d after the start of culture (Fig. 4A; Supplementary Table S1). Thin sections of *35S:BBM* seedlings showed that the embryogenic cotyledon margins comprises a few layers of small, isodiametric cells that were smaller than those in the underlying explant (Fig. 4D). Somatic embryos developed a few days later from this tissue, as single embryos (Fig. 4B) or groups of embryos that were fused at the cotyledons (Fig. 4C). Embryogenic centres formed at the cotyledon margins (Fig. 4E) and produced somatic embryos composed of organs and tissues typical for zygotic embryos (Fig. 4F). The *WOX2:NLS-YFP* embryo marker was expressed in the embryogenic cotyledon margin and developing somatic embryos (Fig. 4G–I).

**Fig. 5.**
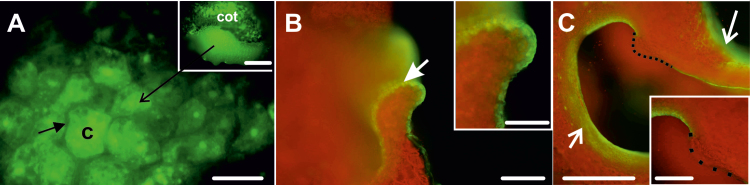
Symplasmic domains during SE in *35S:BBM* seedling explants are characterized by a higher SEL than IZE explants. (A) The surface of a 4-day-old *35S:BBM* seedling cotyledon showing HPTS fluorescence inside the cells (c, cytoplasm; arrow shows absence of the fluorochrome in the cell wall, indicating that its moves between cells through PD). The inset is a lower magnification of the seedling cotyledon (cot). The arrow points to an embryogenic protrusion. (B) F-dextran of 3 kDa applied to the cotyledon margin cells after 4 d of culture (arrow) does not move into other regions of the explant (inset, higher magnification). (C) After 7 d of culture F-dextran fluorescence is visible in the cotyledon margin cells, with the exception of a small group of cells forming an embryogenic protrusion (black dotted line). The arrows mark the site of F-dextran application. Scale bars: (A) 20 µm; (A inset, B, C inset) 100 µm; (B inset) 50 µm; (C) 200 µm.

Symplasmic communication in *35S:BBM* seedlings was analysed with particular emphasis on the cotyledons, the regions where somatic embryos develop. Fluorochrome distribution was studied at sequential stages of development using HPTS. Unlike in WT 2,4-D- or BBM-induced IZE explants, HPTS was observed throughout the cotyledons of *35S:BBM* seedlings regardless of where it was applied and the culture duration (Fig. 5A, inset). This observation prompted us to determine whether the embryogenic and non-embryogenic regions of *35S:BBM* seedlings show differences in the PD SEL (the size of the largest molecules that can diffuse through PD). We examined the pattern of symplasmic movement using 3 kDa F-dextran, which has a higher molecular mass than HPTS and CMNB-caged fluorescein. When F-dextran was applied to the cotyledon margin, the area where somatic embryos are formed, it moved within the cells of the margin, but not to the centre or deeper layers of the cotyledon (Fig. 5B, inset) indicating that the PD SEL of *35S::BBM* seedlings is larger than that of *35S::BBM* IZE explants. F-dextran did not move into embryogenic protrusions, which are the source of somatic embryos, suggesting that two temporally and symplasmically isolated embryogenic domains with different SEL are present within the cotyledon margin of the seedling: a larger domain with SEL ≥3 kDa in embryogenic tissue, and a second domain with the SEL <3 kDa, where multicellular embryos initiate (Fig. 5C, inset).

The data suggest that the movement of low-molecular-mass fluorochromes (CMNB and HPTS) in IZEs (WT and *35S:BBM*) was very limited, regardless of the direction of movement, i.e. from embryogenic to non-embryogenic areas or vice versa, while no movement in any direction was observed for high-molecular-mass dextran. Differences in fluorochrome movement were observed between WT/*35S:BBM* IZEs and *35S:BBM* seedlings; low-molecular-mass fluorochromes moved freely in seedlings (in both directions) while there was very little movement of high-molecular-mass dextran (Table 1).

Summarizing the above, it can be concluded that embryogenic areas are symplasmically isolated from non-embryogenic areas regardless of the explant (IZE or seedling) or inducer treatment (2,4-D or BBM), but with differences in PD SEL (Table 1) between IZE and seedling explants. Notably, embryogenic regions of *35S:BBM* seedlings comprise two symplasmically isolated domains, corresponding to subareas of early embryo growth contained in a larger area of embryogenic cells. In the SE systems studied here, somatic embryos derive from the adaxial protoderm (WT IZEs) or from adaxial protoderm and subprotodermal cells (*35S:BBM* explants). Cell proliferation does take place in other cell layers (Kurczyńska *et al.*, 2007), but it is not known whether these proliferating tissues have a role in direct somatic embryo formation through cell non-autonomous signalling from these underlying, non-embryogenic cells.

### Plasmodesmata between cells following different developmental programmes

**Fig. 6.**
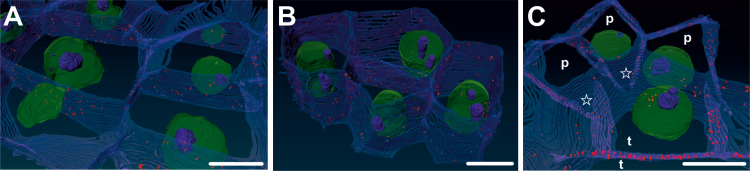
3-D visualization of PD distribution in *35S:BBM* IZE explants. (A) PD distribution between totipotent cells. (B) PD distribution between pluripotent cells. (C) PD distribution on the border between pluripotent (p) and totipotent (t) cells. Stars mark cell walls with lower number of PD on the border pluripotent (p) and totipotent (t) cells; PD, red; nucleus, green; nucleoli, blue. Scale bars: 5 µm.

Accurate and precise determination of PD number and localization within each cell wall is difficult to determine using classical transition electron microscopy because many conditions must be met to obtain a reliable picture of the spatial distribution of PD and their numbers (Zhu and Rost, 2000; Sowiński *et al.*, 2003; Schubert *et al.*, 2013). The major difficulties include collection of successive sections and the unfavourable position of PD on the electron microscopy grid. To overcome these limitations, we used AT analysis (Belevich *et al.*, 2016) for visualization of PD (Fig. 6). AT analysis is a new high-throughput imaging method for high-resolution imaging of tissue ultrastructural architectures (Belevich *et al.*, 2016). This method was used to determine the number of PD and their spatial distribution within the cell walls. Here we used AT analysis to construct a 3-D model of PD distribution in *35S:BBM* IZE explants. *35S:BBM* IZE were chosen due to the abundant production of somatic embryos and the clear separation of symplasmic domains in this material. We constructed the 3-D model of PD distribution between cells with the same identity, i.e. totipotent or pluripotent, as defined by Verdeil *et al.* (2007), and on the border between cells of different phenotypes, i.e. totipotent and pluripotent. The average number of PD was different between the different types of adjacent cells and was the highest in walls between adjacent totipotent cells (121.5±11 SD), followed by adjacent pluripotent cells (78.1±10.3 SD), and juxtaposed totipotent/pluripotent cells (38.9±2.7 SD) (Supplementary Table S2). Thus, the number of PD within and between symplasmic domains differs depending on the developmental fate of the cell.

### Callose biosynthesis precedes and is required for somatic embryo induction

**Fig. 7.**
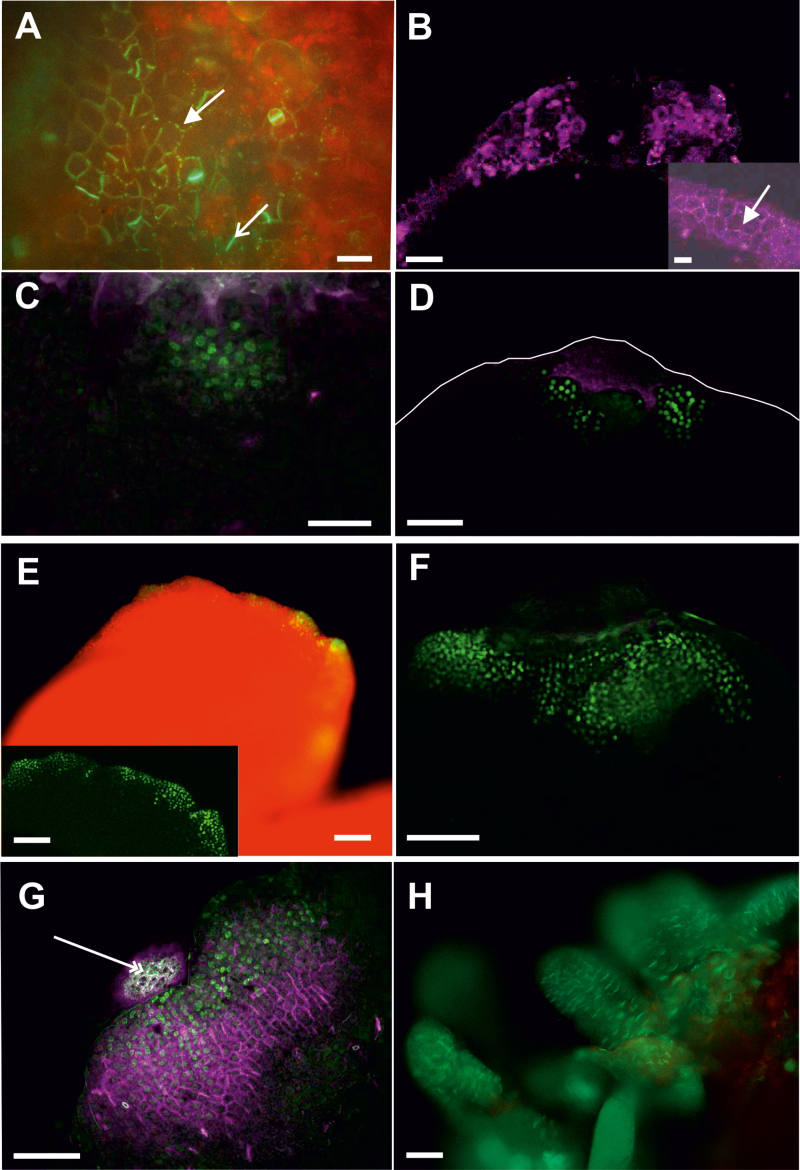
Callose deposition precedes *WOX2* gene expression during *35S:BBM*-induced somatic embryogenesis. (A) Callose staining (green) on the cotyledon tip in 2-day-old *35S:BBM* seedlings. Callose is present in PD located in the primary pit fields (arrow) and in the cell plates of newly divided stomatal meristemoids (open arrow). *WOX2:NLS-YFP* expression was not detected at this stage. (B) Callose staining (purple) along the cotyledon tip and margin in 4-day-old *35S:BBM* seedlings. Inset, higher magnification showing callose (arrow). *WOX2:NLS-YFP* expression was not detected at this stage. (C, D) Five-day-old (C) and 6-day-old (D) *35S:BBM* seedlings showing *WOX2:NLS-YFP* expression (green) and callose staining (purple) in non-overlapping regions. The cotyledon border is marked by a white line. (E, F) Overview of an 8-day-old seedling explant showing *WOX2:NLS-YFP* expression (green) at the cotyledon margin (E; inset optical section showing gene expression) and tip (F). PD callose (purple) is not detected at this stage in the embryogenic region. (G, H) Callose (purple) and *WOX2:NLS-YFP* expression (green) colocalize in the same cells as embryogenic protrusions increase in size (G, 10 d old) and differentiate into somatic embryos (H, 12 d old). Scale bars: (A) 30 µm; (D, E inset, F, G) 50 µm; (B, B inset, C) 20 µm; (E, H) 5 mm.

Our results suggest that embryogenic and non-embryogenic regions of explants are symplasmically isolated during somatic embryo induction. We determined the relationship between these domains and callose deposition in *35S:BBM* seedling explants by following the site and timing of callose deposition in relation to in *WOX2:NLS-YFP* expression. Callose deposition was first observed on the second day of culture, at the tip of the cotyledon and later along the cotyledon margin (Fig. 7A, B). Callose accumulated in the PD in primary pit fields and in stomatal meristemoids. *WOX2:NLS-YFP* expression was only observed from the fifth day of culture onward. Notably, at this time, WOX2-YFP fusion protein and callose were observed in largely mutually exclusive areas, with callose mainly localizing distally to *WOX2* expression at the cotyledon tip and margin (Fig. 7B–D). *WOX2:NLS-YFP* continued to be expressed in low callose/callose-free regions as embryogenic protrusions grew in size (Fig. 7E, F), but by the 10th day of culture, both WOX2–YFP protein and callose began to accumulate in the same cells (Fig. 7G, H). At this stage, callose was mainly localized to the newly formed cell plate (Fig. 7H). Thus, callose deposition at PD initially precedes the establishment of embryo identity, becomes excluded from or reduced in embryogenic protrusions, and then is expressed in newly formed cell walls as embryogenic protrusions increase in size and differentiate. Statistical analysis showed that these developmental steps were highly reproducible between different seedling explants (Supplementary Table S3A). This dynamic regulation of callose biosynthesis is in line with our observations on the presence of embryogenic symplasmic domains.

**Fig. 8.**
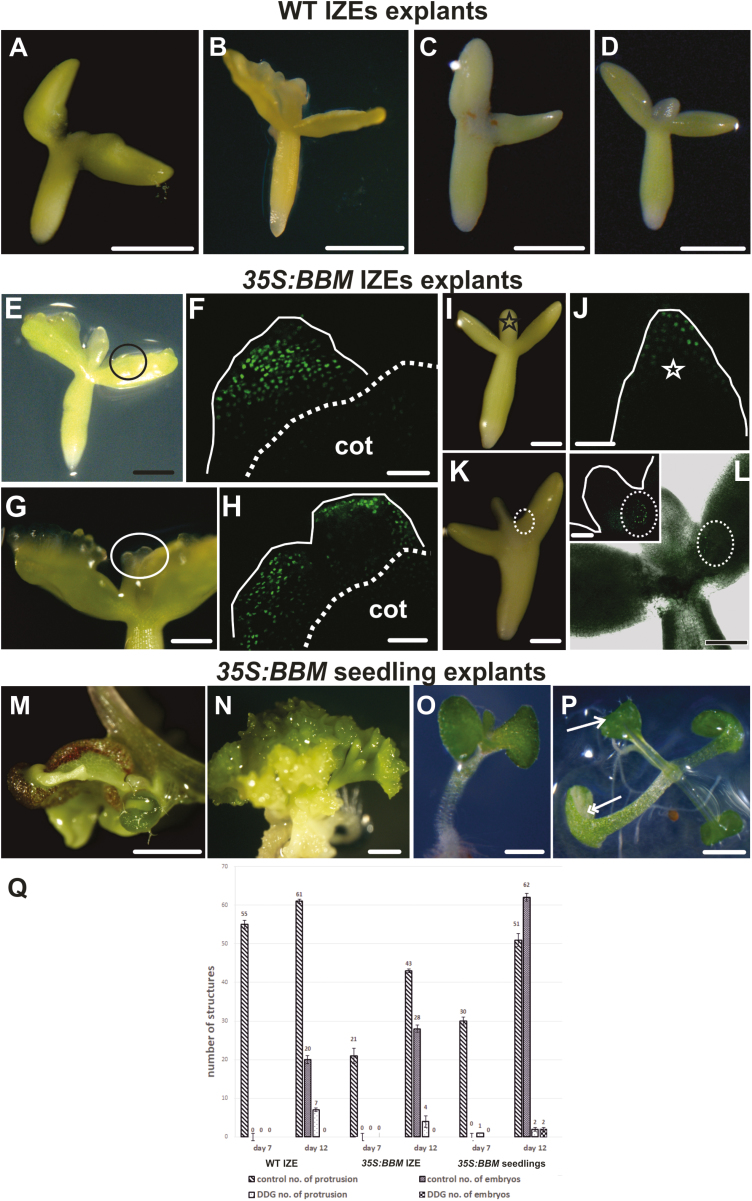
Inhibition of callose biosynthesis suppresses somatic embryo induction. (A–D) Control (A, B) and DDG-treated (C, D) WT IZE explants after 7 d (A, C) and 12 d (B, D) of culture. (E–H) Control *35S:BBM* IZE explants after 7 d (E) and 12 d (G) of culture. (F) *WOX2:NLS-YFP* expression in the same area is shown in (E). The black circle in (E) marks a part of the explant with embryogenic protrusions. (H) *WOX2:NLS-YFP* expression in the same area as shown in (G). The white ellipse in (G) marks the somatic embryos. Dotted lines in (F) and (H) demarcate the areas engaged (above) and not engaged (below) in SE and the white line outlines the explant surface. (I–L) DDG-treated *35S:BBM* explants after 7 d (I) and 12 d (K) of culture. Embryogenic protrusions were greatly reduced and somatic embryo formation was not observed after DDG treatment (I, K). (J, L) *WOX2:NLS-YFP* expression was either limited to a few cells of the explant in the shoot apical meristem, marked by the black star in (I) and white star in (J) and the cotyledon node (marked by the white dotted ellipse in K, L) or absent in all other parts of explants. (M, N) Control *35S:BBM* seedling explants after 7 d (M) and 12 d (N) of culture showing well developed protrusions and somatic embryos. (O, P) Somatic embryogenesis is greatly suppressed in *35S:BBM* seedling explants treated with DDG for 7 d (O) followed by an additional 5 d of culture on DDG-free medium (P) (single arrow marks the leaf, double arrows marks the cotyledons). (Q) SE cultures were treated for 12 d with 0.1 µM DDG and then scored on the indicated days for embryogenic growth (protrusions or somatic embryos). SE, standard error. The differences between means of control and DDG-treated replicates were compared using Dunnett’s test at *P* value<0.05. Scale bars: (A–D, M, N) 500 µm; (E, I, K, L) 200 µm; (F, H, J, L inset) 50 µm; (G) 100 µm; (O–P) 2 mm.

We used the callose biosynthesis inhibitor DDG to determine whether the restriction of PD transport by callose deposition is important for SE induction. IZE explants (WT and *35S:BBM*) and *35S:BBM* seedlings were treated with DDG for 7 d and then transferred to DDG-free medium for an additional 5 d. In all explants, DDG inhibited somatic embryogenesis compared with the non-treated controls (Fig. 8; Table 2). In WT IZE control explants, embryogenic protrusions were abundant after 7 d of culture (Fig. 8A, B), and somatic embryos were well developed after 12 d of culture (Fig. 8Q). In DDG treated explants protrusions were only observed in a few explants after 12 d of culture (Fig. 8C, D, Q). Control *35S:BBM* IZE explants that were grown on medium without DDG developed embryogenic protrusions within 5–7 d of culture and somatic embryos were clearly visible by the 12th day of culture (Table 2; Fig. 8E–H, Q). By contrast, *35S:BBM* IZE explants treated with DDG for the first 7 d of the culture did not develop embryogenic regions on the cotyledon, even after transfer to DDG-free medium. (Fig. 8I, K, Q), although weak *WOX2* expression was observed in a few cells of the cotyledon node and in the shoot apical meristem (Fig. 8J, L). *35S:BBM* seedlings treated with DDG also developed fewer embryogenic protrusions and somatic embryos in comparison with control seedlings (Fig. 8L–P, Q). These results are highly reproducible (Table 2) and show that callose biosynthesis is required for SE. Our results suggest that inhibition of callose biosynthesis prevents the establishment of embryogenic symplasmic domains.

**Table 2.** Inhibition of callose biosynthesis by DDG inhibits somatic embryogenesis and *WOX2* gene expression

Days of culture	Type of explant	WT IZE		*35S:BBM* IZE		*35S:BBM* seedlings	
	Treatment	Control	DDG	Control	DDG	Control	DDG
7	No. of explants	60	60	60	60	66	65
	No. of protrusions/explant	0.9±0.6	0	0.78±0.7	0	0.83±0.7	0
	No. of protrusions expressing *WOX2* /explant	0.88±0.7	0	0.75±0.5	0	0.84 ±0.7	0
	No. of somatic embryos/explant	0	0	0	0	0.03±0.2	0
12	No. of explants	50	50	60	60	66	66
	No. of protrusions/explant	2.2±0.8	0.14±0.2	1.8±1	0.1±0.2	1.9±0.9	0.12±0.3
	No. of protrusions expressing *WOX2*/explant	2.16±0.8	0	1.8±0.8	0.1±0.2	1.63±0.9	0.06±0.2
	No. of somatic embryos/explant	0.9±0.7	0	0.81±0.7	0	1±0.8	0.06±0.2

The data are mean ±SD of three replicates. Note that not all embryogenic protrusions will form embryos and that of the protrusions that make embryos, some will develop more than one embryo. A *z*-test was used to determine significance between proportions in control and DDG-treated cultures for each parameter measured on a given day of culture within a given explant type, i.e. (i) the number of protrusions in the control versus the DDG treatment, (ii) the number of protrusions expressing *WOX2* in the control versus the DDG treatment, and (iii) the number of somatic embryos in the control versus the DDG treatment. The results obtained on day 7 and 12 of the culture and the results in the different explant types were not compared with each other. The control and DDG-treated samples were considered to be statistically significantly different at *P*<0.05 for all of the indicated comparisons.

### Embryogenic protrusions develop in callose-free regions with low DR5 activity

The plant hormone auxin, either in its naturally occurring or synthetic forms, is used extensively to induce SE. Auxin biosynthesis, signalling, and transport genes have also been shown to be direct (transcriptional) targets of the somatic embryo-inducing BBM and LEAFY COTYLEDON1 (LEC1) and LEC2 transcription factors (reviewed in Horstman *et al.*, 2017*a*), although a direct role for the auxin pathway in BBM/LEC-induced SE has not be shown. We therefore examined whether changes in callose accumulation are associated with changes in auxin response during Arabidopsis SE using a post-translationally inducible *35S:BBM-GR* line (Horstman *et al.*, 2017*b*) expressing the auxin response reporter *DR5v2:tdTomato* (Liao *et al.*, 2015).

**Fig. 9.**
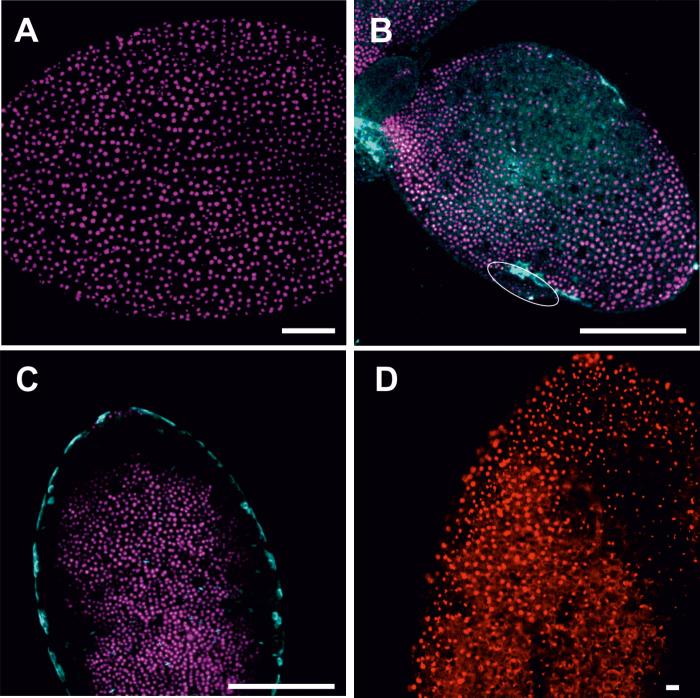
Callose deposition is associated with a decreased *DR5v2* auxin response in embryogenic tissue. DEX-treated *35S:BBM-GR DR5v2:tdTomato* explants were examined for *DR5v2* expression (purple) and callose (blue) in control cultures (A–C) and *DR5v2* expression (red) and callose (blue) in DDG-treated cultures (D). (A) Explants on day 4 of culture showing *DR5* expression on the cotyledon surface. (B) Explant at day 6, showing reduced *DR5v2* expression in patches along the cotyledon surface and margin. Callose begins to accumulate in areas with low *DR5v2* expression. (C) More advanced explant at day 6 showing callose deposition in the cotyledon margin, distal to a region of reduced *DR5v2* expression. (D) *DR5v2* expression is maintained throughout the cotyledon surface after treatment with DDG for 6 d. (A–C) are CLSM and (D) epifluorescence microscopy images. Scale bars: (A–C) 50 µm; (D) 10 µm.

*35S:BBM-GR DR5v2:tdTomato* seedlings were cultured continuously with dexamethasone to induce cytoplasmic to nuclear migration of the BBM–GR protein (Liao *et al.*, 2015) and monitored from day 1 to 7 for *DR5v2* expression and callose deposition. The timing of somatic embryo induction is slower in *35S:BBM-GR* lines than in *35S:BBM* lines, and somatic embryos initially form on the cotyledon margin, rather than the tip. *DR5v2* expression was observed throughout on the adaxial cotyledon surface in 4-day-old DEX-treated *35S:BBM-GR* seedlings (Fig. 9A), and then gradually decreased in patches on the cotyledon surface and the embryogenic cotyledon margin (Fig. 9B), until it was no longer expressed along the margin (Fig. 9C). Callose accumulation was not observed in 4-day-old DEX seedlings (Fig. 9A), but callose began to accumulate in a patchy pattern at the same time that *DR5v2* expression decreased in the same areas (Fig. 9B). Callose accumulation was gradually restricted to the cotyledon margin, distal to the region where embryogenic protrusions develop (Fig. 9C). DDG treatment completely blocked somatic embryo formation, as well as the observed decrease of *DR5v2* expression in the cotyledon margin (Fig. 9D). These data suggest the following developmental steps with respect to auxin response, callose accumulation and embryo initiation: (i) *DR5v2* is initially expressed throughout the cotyledon; (ii) next *Dr5v2* expression decreases and callose appears; and (iii) finally, somatic embryos develop in regions of low *DR5v2* activity. Statistical analysis showed that these developmental steps were highly reproducible between different explants (Supplementary Table S3B).

## Discussion

In most cases, the explants used for somatic embryo induction comprise a complex mixture of tissues and organs that undergo different cell fate changes during culture, such that both embryogenic cell types and a range of non-embryogenic cell types (from pluripotent to differentiated) can be found in the same explant (Boutilier *et al.*, 2002; Raghavan, 2004; Bassuner *et al.*, 2007; de Almeida *et al.*, 2012; Rocha *et al*., 2012, 2015). How individual cells in these explants respond to the different inducer treatments to (re)initiate and maintain totipotent growth is a major unanswered question in the field. Here we show, using three different Arabidopsis SE systems, that symplasmic isolation of embryogenic cells from non-embryogenic cells is a major driver of this process.

### Establishment of symplasmic domains marks somatic embryo initiation

Symplasmic communication plays an important role in regulating the movement of various types of signalling molecules between cells (Marzec and Kurczynska, 2014; Tilsner *et al.*, 2016) and disruption of the normal symplasmic communication pattern leads to changes in plant growth (for review see Marzec and Kurczynska, 2014; Lu *et al.*, 2018). Symplasmic isolation often precedes or occurs simultaneously with the initiation of cell differentiation, suggesting that it is required for cell differentiation (Tilsner *et al.*, 2016). We show that symplasmic communication also changes during the course of somatic embryo induction, from well-established symplasmic communication between all explant cells, to the establishment of symplasmic subdomains in regions undergoing different developmental fates.

After 1 d of culture 2,4-D-treated WT IZE explants and *35S:BBM* IZE explants comprised a single symplasmic domain with respect to the small tracers like HPTS and fluorescein. However, this communication became limited during the course of culture as regions of the explants switched to embryogenic growth. Symplasmic subdomains were established on the adaxial surface of the WT and *35S:BBM* IZE cotyledons around the fifth to sixth day of culture (Fig. 2, 3). The timing and location of this symplasmic domain establishment corresponded to the timing and location of somatic embryo initiation at the histological level (Kurczyńska *et al.*, 2007; Kulinska-Lukaszek *et al.*, 2012). Moreover, the spatial localization of *WOX2* gene expression was highly correlated with those areas of the explant that were symplasmically isolated.

Symplasmic domains corresponding to embryogenic and non-embryogenic cells were also established in *35S:BBM* seedlings, but with a higher PD SEL than those in IZE explants. Somatic embryos formed along the adaxial cotyledon surface of *35S:BBM* IZE explants, while in *35S:BBM* seedlings, embryos formed on the cotyledon margin. In WT Arabidopsis seedlings PD have distinct SELs in different subdomains including the shoot apical meristem, but no symplasmic domain has been described at the cotyledon margin (Kim *et al.*, 2005). This suggests that during somatic embryo induction, symplasmic domains develop *de novo* and separate embryogenic and non-embryogenic cells.

Cell differentiation is correlated with the formation of symplasmic domains, and the more advanced the state of cell differentiation the lower the symplasmic communication (Kim and Zambryski, 2005; Kobayashi *et al.*, 2007; Faulkner, 2018). Our results show that symplasmic isolation is established between cells realizing different developmental programmes in somatic embryo culture, i.e. embryogenic (totipotent) and meristematic (pluripotent) (Fig. 1). These symplasmic domains are established regardless of the inducer treatment (2,4-D or BBM) or the explant (IZEs or seedlings), indicating that it is a shared response during the (re)initiation of totipotent growth from different Arabidopsis explants (Figs 2–4).

Cell differentiation is correlated with the formation of symplasmic domains, and the more advanced the state of cell differentiation, the lower the symplasmic communication (Kim and Zambryski, 2005; Kobayashi *et al.*, 2007; Faulkner, 2018). Our results show that symplasmic isolation is established between cells realizing different developmental programmes in somatic embryo culture, i.e. embryogenic (totipotent) and non-embryogenic (meristematic/pluripotent or differentiated). These symplasmic domains are established regardless of the inducer treatment (2,4-D or BBM) or the explant (IZEs or seedlings), suggesting that it is a shared response during the (re)initiation of totipotent growth from multicellular Arabidopsis explants.

Symplasmic communication/isolation also plays a role in cell-to-cell communication and differentiation during normal plant development (Duckett *et al.*, 1994; Oparka *et al.*, 1994; Kim and Zambryski, 2005). Thus, it is likely that symplasmic communication/isolation is not restricted to embryogenic areas, but also take place in areas of the explant that are not involved in SE. While we have not observed movement of low-molecular-mass fluorochromes from the subprotodermal cells into the protodermal cells, we cannot state that symplasmic communication is completely restricted between these two areas, as molecules with a smaller size or Stoke’s radius should be able to move freely through plasmodesmata in a non-targeted manner by diffusion or following electrochemical gradients (Imlau *et al.*, 1999; Oparka *et al.*, 1999; Zambryski and Crawford, 2000; Wu *et al.* 2003).

### Embryogenic regions in different explants have different size exclusion limit of plasmodesmata

**Fig. 10.**
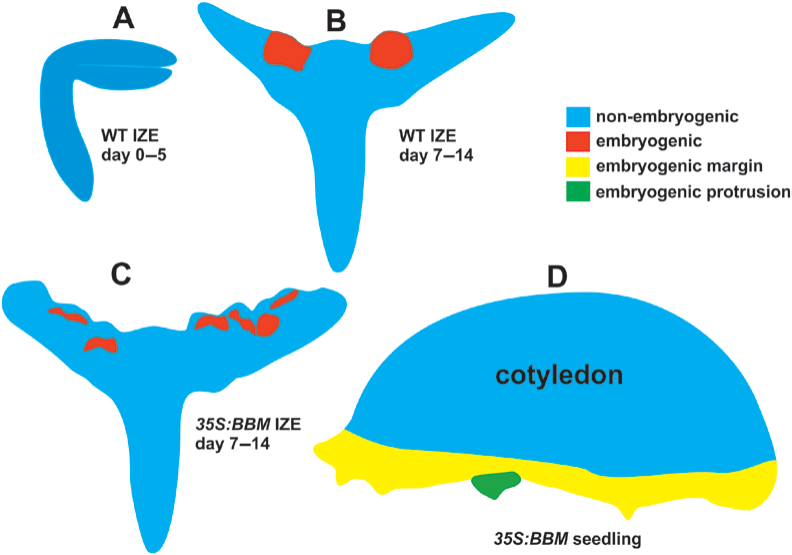
Schematic diagram showing the symplasmic domains found in different explants during somatic embryo culture. (A) IZE explants (representative for both WT and *35S:BBM* IZEs) at the start and during the first day of culture; the entire explant is a single symplasmic domain for low-molecular-mass fluorochromes up to the sixth day of the culture. (B, C) Two symplasmic domains are detected for low-molecular-mass fluorochromes in WT IZE explants (B) and *35S:BBM IZE* explants (C) between the sixth and eighth day of the culture, corresponding to an embryogenic region (red) and a non-embryogenic region (blue). (D) Symplasmic domains for 3 kDa tracers in *35S:BBM* seedling explants. The three symplasmic domains are the non-embryogenic region (blue), the embryogenic cotyledon margin (yellow) in which 3 kDa dextran was retained, and the region with embryogenic protrusions (green), which is unable to take up 3 kDa dextran.

Differences in SEL between symplasmic domains restricts communication between cells in these different domains, thus enabling the initiation of specific developmental programmes at the cell, tissue, and organ levels (Kim and Zambryski, 2005; Sevilem *et al.*, 2015; Tilsner *et al.*, 2016). Small molecules such as metabolites, including sugars and amino acids, as well as hormones are thought to move through PD by a non-targeted diffusive mechanism (Wu *et al.*, 2003), while larger molecules such as proteins, including transcription factors, move by both targeted and non-targeted mechanisms (Burch-Smith *et al.*, 2011). Our results indicate that the PD SEL of IZE explants is about 0.5 kDa, while the PD SEL of *35S:BBM* seedling explants is *ca*. 3 kDa in the embryogenic region that is initially established on the cotyledon margin, and less than 3 kDa in the embryogenic centres that develop within this margin (Fig. 10).

The intercellular movement of molecules through PD is based on their molecular mass, as well as their shape and effective Stokes radius (Terry and Robards, 1987), such that molecules with a lower molecular mass might have a larger diameter than the molecules of larger molecular mass (Marzec and Kurczynska, 2014). Based on the tracers used in this study, we estimate that the diameter or molecular exclusion limit of PD on the border between the embryogenic and non-embryogenic WT IZE explant areas is less than 0.9 nm, and about 1.2 nm on the border between the embryogenic and non-embryogenic cotyledon regions in *35S:BBM* seedling explants. The molecular exclusion limit on the border between the embryogenic protrusions and the rest of embryogenic margin in BBM seedling explants is smaller than 1.2 nm, as F-dextran did not cross this boundary, but this needs to be better defined with additional lower molecular mass fluorochromes. These data suggest that embryogenic protrusions are isolated from the other embryogenic parts of the *35S:BBM* seedling explants by a smaller SEL value that is similar to that of 2,4-D-treated and BBM-induced IZE explants (Fig. 5). Thus, the formation of embryogenic cells and their further growth into histodifferentiated embryos is associated with a significant limitation of the movement of molecules through PD.

It is surprising that two different PD SELs are established in embryogenic tissues of IZE and seedling explants. The main developmental difference is that IZE explants already possess embryo identity, while seedling explants need to re-establish embryo identity. We propose that there is a one-step mechanism for somatic embryo initiation in IZEs, while in seedlings a two-step mechanism is required. In both explants, the cotyledon cells (re)establish embryogenic growth in a separate symplasmic domain. This domain is sufficient to direct further embryo growth and differentiation in IZEs, but in seedlings a second sub-symplasmic domain with a smaller SEL is needed to promote further embryo development.

The low PD SEL between embryogenic and non-embryogenic explant domains is similar to the low PD SELs observed during zygotic embryogenesis (Han *et al.*, 2000), the onset of flowering (Burch-Smith *et al.*, 2011), and for stem cell maintenances in the shoot apical meristem (Rinne and van der Shoot, 1998). This low SEL value allows ions (Erwee and Goodwin, 1985), organic acids (Spanswick, 1976), carbohydrates (Botha and Black, 2000; Knoblauch and Peters, 2013), and hormones (Maule *et al.*, 2011; Han and Kim, 2016) to move freely through PD, but restricts protein movement (Lucas and Lee, 2004). In this respect, auxin is an interesting candidate for a symplasmically restricted signal, given its role in driving induced cell totipotency. Somatic embryogenesis is induced by (synthetic) auxins, which in turn induce expression of somatic embryo-promoting transcription factor genes and endogenous auxin biosynthesis genes (Ledwoń and Gaj, 2009; Bai *et al.*, 2013; Fehér, 2015). Likewise, somatic embryo-promoting transcription factors like BBM/AILs, LEC1, and LEC2 bind and transcriptionally regulate auxin biosynthesis, signalling, and transport genes (Braybrook *et al.*, 2006; Junker *et al.*, 2012; Horstman *et al*., 2017*a*,*b*), although a direct role for auxin in BBM/LEC-induced SE has not been shown. Han *et al.* (2014) described an auxin–callose feedback loop in which closed PD promote efficient development of an auxin gradient by preventing diffusion of auxin back into the cell through open PD. Although the natural auxin indole acetic acid has a small molecular mass of about 200 Da, the calculated Stokes radius is 3.2 nm (Grigolon *et al.*, 2015), which is larger than the estimated PD SEL of embryogenic explant domains in somatic embryo cultures. It is therefore possible that the symplasmic transport of auxin or specific auxin-related mRNAs or proteins is restricted in embryogenic domains during SE (Fig. 9).

### Distribution of plasmodesmata correlates with cell phenotype

Knowledge of complex 3-D structures of cells and cell organelles in their natural context is important for understanding the structure–function relationship (Belevich *et al.*, 2016). Such models are increasingly being developed for animal cells (Briggman and Bock, 2012; Wacker *et al.*, 2015; Russell *et al.*, 2017). In plant cells, 3D reconstructions have been described for a few plants, including Arabidopsis (Furuta *et al.*, 2014; Płachno *et al*., 2017; Zechmann and Zellnig, 2017; Reagan *et al.*, 2018). A number of studies have examined PD number in different cells of the same tissue, for example in vascular tissues (Sowiński *et al.*, 2003) and roots (Gunning *et al.*, 1978; Zhu and Rost, 2000; Schubert *et al.*, 2013), but to the best of our knowledge, a 3-D reconstruction of PD number and distribution between adjacent cells following the same or a different developmental programme has not been presented (Fig. 6).

In addition to PD SEL, the shape, number, and distribution of PD are developmentally regulated (Rutschow *et al.*, 2011; Marzec and Kurczynska, 2014). Our results show that there are more PD between adjacent cells following the same developmental programme (totipotent–totipotent and pluripotent–pluripotent) compared with cells following different development programs (totipotent–pluripotent). This result is consistent with observations showing that the PD number is different in embryogenic and non-embryogenic cells (Emons, 1994; Jasik *et al.*, 1995). This implies that the abundant plasmodesmal connections between cells realizing the same developmental programme reflects the importance of intercellular communication and coordination between these cells (Jasik *et al.*, 1995), and, on the other hand, limitation of symplasmic communication on the border between cells realizing different developmental programmes blocks the movement of signals between different symplasmic domains enabling the implementation of different development programmes. The presence of PD between embryogenic and non-embryogenic regions of the same explant provides further support for the observed changes in symplasmic communication between these regions being the result of changes in PD permeability and not the absence of PD.

### Callose deposition at plasmodesmata is required for establishment of *in vitro* totipotency in Arabidopsis

Here we show that callose deposition precedes the establishment of embryo identity, and later, that these embryogenic regions show reduced callose accumulation (Figs 7, 8). Chemically inhibiting callose biosynthesis results in the loss of embryo identity in somatic embryo cultures derived from two different explants induced by two treatments. These data suggest that a reduction in PD SEL by callose deposition, and the associated changes in symplasmic communication (Dubois *et al*., 1990, 1991; Puigderrajols *et al.*, 2001; You *et al.*, 2006), are a general requirement for the establishment of totipotency in tissue culture. Symplasmic isolation between explant and embryogenic regions during SE might be analogous to the symplasmic isolation of the zygote and the maternal tissues during zygotic embryogenesis in the seed (Yeung *et al.*, 1996). In both systems, symplasmic isolation from surrounding tissues might serve to reinforce cell fate specification.

## Supplementary data

Supplementary data are available at *JXB* online.

Table S1. Timetable of morphogenic events and *WOX2:NLS-YFP (WOX2)* gene expression during SE from different explants.

Table S2. The number of plasmodesmata between cells in *35S:BBM IZE* explants depends on the developmental fate of the cell.

Table S3. Quantitative analysis of callose deposition and gene expression in *35S:BBM* seedling explants.

eraa041_suppl_Supplementary_Tables_S1-S3

## Abbreviations:

AT: array tomography
BBM: BABY BOOM
CMNB: bis-(5-carboxymethoxy-2-nitrobenzyl)
DDG: 2-deoxy-D-glucose
HPTS: 8-hydroxypyrene-1,3,6-trisulphonic acid
IZE: immature zygotic embryo
PD: plasmodesmata
SE: somatic embryogenesis
SEL: size exclusion limit
WOX2: WUSCHEL-RELATED HOMEOBOX 2
