# Supplementary material for: Symplasmic isolation marks cell fate changes during somatic embryogenesis
Source: J Exp Bot. 2020 Jan 24;71(9):2612–28. doi: 10.1093/jxb/eraa041 (PMC7210756; doi:10.1093/jxb/eraa041)
Supplement: eraa041_suppl_Supplementary_Tables_S1-S3 [file eraa041_suppl_supplementary_tables_s1-s3.pdf]

Supplementary Table S1. Time-table of morphogenic events and *WOX2:NLS-YFP* (*WOX2*) gene expression during SE from different explants.

Culture duration (days)	0-7	8-14	15-21	22-30
WT IZE	Day 5: *WOX2* expression in adaxial proximal part of cotyledons. Day 6: embryogenic (*WOX2*-expressing) protrusions on adaxial cotyledon.	Continued embryogenic protrusion development. Globular stage somatic embryos.	Somatic embryos at different stages of development.	Increased numbers of somatic embryos, both primary and secondary.
35S:BBM IZE	Day 3: *WOX2* expression on adaxial cotyledon. Day 4: embryogenic (*WOX2*-expressing) protrusions on adaxial cotyledon.	Continued embryogenic protrusion development. Somatic embryos in early stages of development.	Somatic embryos at different stages of development.	Secondary somatic embryos.
35S:BBM seedlings	Day 5: *WOX2* expression on the cotyledon margin. Day 6: *WOX2*-expressingin embryogenic growth protrusions on the cotyledon margin.	Continued embryogenic protrusion development. Somatic embryos at different stages of development. Secondary somatic embryo development.	Increased number of somatic embryos, both primary and secondary.	Increased number of somatic embryos.

Supplementary Table S2. The number of plasmodesmata between cells in *35S:BBM* IZE explants depends on the developmental fate of each cell.

35S:BBM IZE explants	Average ±SD
totipotent/totipotent	121.5^{a}±11
pluripotent/pluripotent	78.1^{b}± 10.3
totipotent/pluripotent	38.9^{c}±2.7

35S:BBM IZE explants were sampled on the fifth day of culture. The average number of plasmodesmata (PD) on the border between cells was counted for adjacent cells showing the same phenotype (totipotent/totipotent or pluripotent/pluripotent) or for adjacent cells showing different phenotypes (totipotent/pluripotent). PD were counted in three independent biological replicates, using five adjacent cells for each phenotype. Statistically significant differences between each determined cell phenotypes were calculated in simultaneous pairwise comparisons using Tukey's HSD (honestly significant difference) test. Different letters indicate statistically significant different values at p=0.05. SD, standard deviation.

Supplementary Table S3. Quantitative analysis of callose deposition and gene expression in *35S:BBM* seedling explants.

Supplementary Table S3A. Quantitative summary of the number of *35S:BBM* seedling explants showing the indicated patterns of callose deposition and *WOX2* expression at different time points in culture.

**Developmental event**	**1** (days 3-5)	**2** (days 5-7)	**3** (days 8-14)
average ± standard deviation	89.4 % ±0.071 (n=38)	88.5 % ±0.046 (n=35)	93.1% ±0.052 (n=29)

Reproducibility of the consecutive developmental events (1-3) in *35S:BBM* seedlings occurring on the indicated days of culture: 1) callose present, no *WOX2* gene expression (as in Fig. 7B); 2) callose present, *WOX2* gene expression present (as in Fig. 7C, D); 3) reduced callose levels, *WOX2* gene expression present (as in Fig. 7F). Embryogenic areas were identified based on their known histological characteristics (see Fig. 4D, E). n, number of seedling explants analysed in three independent biological replicates. The percentage of explants realizing developmental events 1 to 3 was calculated as follows: (number of explants realizing the indicated pathway/n) x 100 %.

Supplementary Table S3B. Quantitative summary of the number of *35S:BBM-GR* seedling explants showing the indicated patterns of callose deposition and *DR5v2* expression at different time points in culture.

**Developmental event**	**1** (day 0-4)	**2** (days 5-7)
average ± standard deviation	85.7 % ± 0.13 (n=14)	76.9 % ±0.025 (n=13)

Reproducibility of the consecutive developmental events (1-2) in DEX-induced *35S:BBM-GR* seedlings occurring on the indicated days of culture: 1) *DR5v2* expressed throughout explant cotyledon, no callose accumulation (as in Fig. 9A); 2) decrease in *DR5v2* expression in embryogenic areas (cotyledon margin), callose accumulates in embryogenic areas (as in Fig. 9B, C). Embryogenic areas were identified based on their known histological characteristics (see Fig. 4D, E). n, number of seedling explants analysed in three independent biological replicates. The percentage of explants realizing developmental events 1 to 2 was calculated as follows: number of explants realizing the indicated pathway/n) x 100 %.
